# A haplotype-like, chromosome-level assembled and annotated genome of *Biomphalaria glabrata*, an important intermediate host of schistosomiasis and the best studied model of schistosomiasis vector snails

**DOI:** 10.1371/journal.pntd.0011983

**Published:** 2024-02-29

**Authors:** Daibin Zhong, Lijing Bu, Mohamed R. Habib, Lijun Lu, Guiyun Yan, Si-Ming Zhang

**Affiliations:** 1 Program in Public Health, College of Health Sciences, University of California, Irvine, California, United States of America; 2 Center for Evolutionary and Theoretical Immunology, Department of Biology, University of New Mexico, Albuquerque, New Mexico, United States of America; University of Glasgow, UNITED KINGDOM

## Abstract

Schistosomiasis is one of the world’s most devastating parasitic diseases, afflicting 251 million people globally. The Neotropical snail *Biomphalaria glabrata* is an important intermediate host of the human blood fluke *Schistosoma mansoni* and a predominant model for schistosomiasis research. To fully exploit this model snail for biomedical research, here we report a haplotype-like, chromosome-level assembled and annotated genome of the homozygous iM line of *B*. *glabrata* that we developed at the University of New Mexico. Using multiple sequencing platforms, including Illumina, PacBio, and Omni-C sequencing, 18 sequence contact matrices representing 18 haploid chromosomes (2n = 36) were generated (337x genome coverage), and 96.5% of the scaffold sequences were anchored to the 18 chromosomes. Protein-coding genes (n = 34,559), non-coding RNAs (n = 2,406), and repetitive elements (42.52% of the genome) were predicted for the whole genome, and detailed annotations for individual chromosomes were also provided. Using this genomic resource, we have investigated the genomic structure and organization of the *Toll-like receptor* (*TLR*) and *fibrinogen-domain containing protein* (*FReD*) genes, the two important immune-related gene families. Notably, *TLR-like* genes are scattered on 13 chromosomes. In contrast, almost all (39 of 40) *fibrinogen-related genes* (*FREPs*) (immunoglobulin superfamily (IgSF) + fibrinogen (FBG)) are clustered within a 5-million nucleotide region on chromosome 13, yielding insight into mechanisms involved in the diversification of *FREPs*. This is the first genome of schistosomiasis vector snails that has been assembled at the chromosome level, annotated, and analyzed. It serves as a valuable resource for a deeper understanding of the biology of vector snails, especially *Biomphalaria* snails.

## Author summary

We present a chromosome-level assembled and annotated genome sequence of the freshwater gastropod snail *Biomphalara glabrata*, offering a comprehensive genomic view of the structure and organization of two crucial immune-related gene families: the *Toll-like receptor (TLR)* and *fibrinogen-domain containing protein (FReD)* genes. *B*. *glabrata* is an important intermediate host of schistosomiasis, a parasitic disease afflicting more than 251 million people worldwide. In addition to offering insight into the diversification of fibrinogen-related protein (*FREP*) genes, the high-quality genome assembly of the model vector snail *B*. *glabrata* provided by this study will enhance our understanding of fundamental biological processes at the molecular level and accelerate snail-based research of schistosomiasis.

## Introduction

Schistosomiasis is one of the world’s most devastating parasitic diseases, caused by blood flukes (trematode parasites) of the genus *Schistosoma* [1]. Approximately 251 million people in 78 countries are affected by schistosomiasis [2], leading to significant morbidity and mortality. Additionally, it plays a role in the transmission of the human immunodeficiency virus (HIV) [3,4], and is associated with the development of bladder cancer [5,6]. The current treatment relies solely on praziquantel (PZQ), a drug that has been used for over 40 years [7]. While PZQ effectively targets against adult schistosome worms, it does not prevent re-infection. PZQ-treated patients, especially children, quickly become re-infected upon contacting waterbodies infested with cercariae—the human-infective stage of the parasite, shed from the snail intermediate hosts [8,9]. Snail control has proven to be one of the most effective means of reducing the prevalence of schistosomiasis in endemic regions [10,11].

Three freshwater molluscan genera, namely *Biomphalaria*, *Bulinus*, and *Oncomelania*, that comprise approximately 73 species (34 *Biomphalaria*, 37 *Bulinus*, and 2 *Oncomelania*) are responsible for vast majority of human schistosomiasis transmission, although not all species are implicated in this transmission. Within the gastropod genus *Biomphalaria*, which is geographically distributed in the Old and New World, 18 species are susceptible to *Schistosoma mansoni* [12,13]. *Biomphalaria glabrata* has been used as a predominant model species for studies of snail-parasite interactions and schistosomiasis since the 1950s [14–17], accumulating a wealth of biological information [18–22]. *B*. *glabrata*, a hermaphrodite with 36 chromosomes (2n = 36, no sex chromosomes) [23], is the most important schistosomiasis vector snail in the New World. Over the past decades, several strains of *B*. *glabrata* have been developed in the laboratory or isolated from the field. The albino M line was selected from early crosses between albino Brazilian and pigmented Puerto Rican snail strains in the 1950s [17]. The pigmented BS90 was isolated from the field in the 1960s in Salvador, Brazil [24]. The 13-16-R1 strain was obtained by crossing highly resistant strains of snails isolated from Brazil and Puerto Rico [25,26]. The BBO2 and Guadeloupe strains were collected from natural populations in Brazil [27] and Guadeloupe [28], respectively. In addition, some research groups have maintained their own laboratory strains [29]. Among all available strains, two strains (M line and BS90) exhibiting two different phenotypes of schistosome resistance have been widely used for research and supplied by the NIH-funded Schistosomiasis Resource Center of Biomedical Research Institute (BRI) for worldwide research (www.afbr-bri.org/schistosomiasis) [30]. The M line is generally susceptible to *Schistosoma mansoni*, whereas BS90 demonstrates resistant to the parasites. Moreover, *B*. *glabrata* is one of the few intensively studied laboratory models in Mollusca, the second-largest animal phylum after Arthropoda. The Bge cell line, derived from embryonic cells of *B*. *glabrata*, is the only existing cell line originating from any molluscan species [31–33].

The complete nuclear genome sequence is essential for understanding the complexity of snail biology, paving the way for the development of innovative snail-targeted biocontrol programs. Given the pivotal role of *B*. *glabrata* in schistosomiasis transmission and research, as described above, *B*. *glabrata* (BBO2 strain) was selected from the three genera of human schistosome-transmitting molluscs for the first genome sequencing. The draft genome was assembled in 2017 by an international consortium comprising 117 researchers from 10 countries [27]. This pioneering reference genome has provided a valuable resource for data mining and genomics studies. However, the genome assembly for this strain is highly fragmented and has limited application because it was derived from 331,400 scaffolds (N50 = 48 Kb).

We have recently published two improved genome sequences from two homozygous lines of *B*. *glabrata* (iM line and iBS90) that we developed at the University of New Mexico (UNM) [34]. These genomes have been instrumental in conducting linkage mapping and genetic analysis related to schistosome resistance and body pigmentation (for detailed information regarding the development of iM line and iBS90 snails, see [34]). Building on these achievements and leveraging the advantages of the unique homozygous iM line of *B*. *glabrata* (81 generations of self-fertilization from a single M line snail resulted from an 18-year breeding effort), we have further enhanced the genome assembly quality. This improvement includes the attainment of a chromosome-level genome assembly, achieved through the incorporation of newly generated Omni-C sequencing data. Omni-C sequencing, a novel iteration of Hi-C sequencing, employs a sequence-independent endonuclease for chromatin digestion before proximity ligation chromosome conformation capture sequencing. This groundbreaking technology utilizes in vivo chromatin proximity information, resulting in significantly improved genome assemblies [35,36].

The genome sequence presented in this paper represents the first comprehensive analysis and report of a chromosome-level assembled and annotated genome within the three molluscan genera of human schistosomiasis vectors. While scaffold-level assembled genomes have been documented for several species of schistosomiasis vector snails, including *B*. *glabrata* [27,34], *B*. *pfeifferi* [37], *B*. *straminea* [38], and *Bulinus truncatus* [39], this work stands out as the first to achieve a chromosome-level assembly for *B*. *glabrata*, as evidenced by its availability in the National Center for Biotechnology Information (NCBI) database (xgBioGlab47.1) (further details can be found in the Discussion section). Furthermore, the high-quality genome provides a platform for an in-depth investigation into the genomic architecture of *Toll-like receptor (TLR)* and *fibrinogen-domain containing protein (FReD)* genes, both crucial components of immune-related gene families [40–46]. This analysis sheds light on the diversification of *fibrinogen-related protein (FREP)* genes in *B*. *glabrata* [47,48].

## Materials and methods

### Snails

Genomic DNA was extracted from the homozygous iM line of *B*. *glabrata* that has been developed and maintained at the University of New Mexico (UNM) [34]. Three sequencing platforms, including Illumina, PacBio (Pacific BioSciences), and Omni-C sequencing, were applied. For each platform, a single iM line of *B*. *glabrata* snail was used for DNA extraction and sequencing library preparation. Illumina sequencing was performed using a single snail resulted from 72 generations of self-fertilization (G72). Two individual snails from 81 generations (G81) of self-fertilization were used for PacBio and Omni-C sequencing, respectively. As the G72 iM line genome was confirmed homozygous at the genome level [34], the three homozygous individuals used for the three sequencings should be genomically identical.

### Short-read Illumina and long-read PacBio sequencing

Illumina and PacBio sequencing were conducted at the Molecular Biology Facility of UNM Biology Department (https://ceti.unm.edu/core-facilities/molecular-biology.html) and DNA Sequencing Center of Bingham Young University (https://lifesciences.byu.edu/dna-sequencing-center-logos), respectively (for details see [34]).

### Omni-C sequencing

For the Omni-C library preparation, a single G81 iM line snail was used for the Omni-C library preparation, to which the Dovetail Omni-C kit was applied. Briefly, the chromatin was fixed with disuccinimidyl glutarate (DSG) and formaldehyde in the nucleus. The crosslinked chromatin was then digested *in situ* with DNase I. Following digestion, the cells were lysed with SDS to extract chromatin fragments, which were subsequently bound to chromatin capture beads. Next, the chromatin ends were repaired and ligated to a biotinylated bridge adapter, followed by proximity ligation of the adapter-containing ends. After proximity ligation, the crosslinks were reversed, the associated proteins were degraded, and the DNA was purified and converted into a sequencing library using Illumina-compatible adaptors. Biotin-containing fragments were isolated using streptavidin beads prior to PCR amplification. The library was sequenced on an Illumina platform to generate paired-end reads (150 bp x 2). Omni-C sequencing was performed by Dovetail (formerly Dovetail Genomics: https://dovetailgenomics.com/; now Cantata Bio: https://cantatabio.com).

### Scaffolding the genome assemblies using HiRise

The de novo assemblies [34] and Dovetail Omni-C reads were served as the input data for scaffolding using HiRise, a software pipeline designed specifically for using proximity ligation data to scaffold genome assemblies [49]. Dovetail Omni-C sequences were aligned to the input draft assembly using BWA (https://github.com/lh3/bwa). The separations of Dovetail Omni-C read pairs mapped within draft scaffolds were analyzed by HiRise to produce a likelihood model for genomic distance between read pairs, and the model was used to identify and break putative misjoins, to score prospective joins, and make joins above a threshold. The threshold is used to enhance the accuracy and reliability of the assembly, guiding the identification and action upon potential joins or breaks in the genomic scaffolds. When the model predicts a high likelihood that a pair of segments is correctly adjacent, HiRise interprets this as a prospective join and proceeds to join these segments. Conversely, segments below this threshold are not joined. Furthermore, the threshold allows scrutinizing existing scaffold connections; connections that fail to meet this criterion are considered misjoins and are broken.

### Prediction of repetitive elements and protein-coding genes

Annotations for repetitive elements and protein-coding genes at the whole-genome level were carried out using the same methodology employed for the assembly of scaffold-level genomes of the iM line and iBS90 of *B*. *glabrata* [34] and *B*. *pfeifferi* [37]. The annotation includes three main steps: 1) identification of repetitive elements throughout genome and soft-mask these repeats (change from upper case to lower case in genome sequences); 2) gene model prediction using repeat soft-masked genome; and 3) functional annotation for the gene models, as described below.

Repetitive sequence analysis was performed using RepeatModeler 2.0.1 [50] with Dfam transposable elements TE Tools v1.2 [51]. To avoid misidentification of genes derived from a large gene family, the predicted repeat models were searched against the InterProScan database [52] to retain models with either no domain or only retrotransposon domains (excluding all functional domains). Finally, the assembly was softly masked with clean repeat models using RepeatMasker 4.062 [53].

Gene models were predicted using EVidence Modeler [54], incorporating weighted evidence from *ab initio* predictions, RNA sequencing (RNAseq) alignments, and sequence similarity-based searches against known transcript sequences. RNAseq data from 12 tissues of *B*. *glabrata* [27] and whole bodies of *B*. *glabrata* exposed or non-exposed to *S*. *mansoni* [55] were used to align to the genome using Program to Assemble Spliced Alignments (PASA) pipeline [56], then fed to Braker2 [57] to obtain reference-based protein-coding gene model prediction evidence, as previously described [34]. The *ab initio* prediction evidence is comprised of core single-copy orthologs identified using BUSCO (Benchmarking Universal Single-Copy Orthologs) customized HMM models built from tBLASTn [58] and AUGUSTUS predictions [59].

Function annotation for the predicated gene models was performed using sequence similarity search. Extracted protein-coding nucleotide (nt) and amino acid (aa) sequences were used as queries to search against four reference databases: 1) BLASTp to the UniProt [60] database with a minimum identity of 30%, minimum aligned length 10 aa, and an E value of 10^−5^; 2) BLASTp to the NCBI non-redundant protein database (NR) with the same cutoff as above; 3) BLASTn to the NCBI non-redundant nucleotide database (NT) with >60% identity and E value10^−5^; 4) search for the conserved functional domain by InterProScan 5.45 [52].

### Prediction of non-coding RNAs

Four major non-coding RNAs, including microRNA (miRNA), long non-coding RNA (lncRNA), transfer RNA (tRNA), and ribosomal RNA (rRNA), were predicted. Sequence profiles containing covariance models (CMs) of known non-coding RNAs were downloaded from the Rfam database and used as queries to search the assembled genome. The search was performed using Infernal software (INFERence of RNA Alignment) [61], with options “cmscan—cpu 40—rfam—cut_ga—nohmmonly—tblout mrum-genome.tblout—fmt 2—clanin Rfam.clanin Rfam.cm”. miRNA sequence similarity identified at the chromosome-level genome of the iM line of *B*. *glabrata* genome was extracted. Additionally, a highly automated pipeline with classified miRNA CMs, named mirMachine pipeline 0.2.11.2, was applied to identify miRNAs with the node set to Mollusca. The miRNAs predicted from the two methods were merged and duplicate records with overlapping locations were removed. The miRNAs of *B*. *glabrata* were grouped using cd-hit-est 4.8.1 [62].

For the annotation of lncRNAs, RNAseq reads of *B*. *glabrata* used for gene model prediction as described above were applied. The reads were initially mapped to the current *B*. *glabrata* chromosome-level genome assembly. The genome-guided transcriptome assembly was based on the alignment of RNAseq reads to the genome using Trinity 2.8.5 [63]. Only sequences longer than 200 nt (nucleotides) were retained after filtration. The protein-coding potential of each transcript sequence was assessed using Coding Potential Calculator version 2 (CPC2) [64]. Potential protein-coding sequences were further filtered by BLASTx search against the NCBI nr database with the options “-f 100—max-target-seqs 20—masking 1—evalue 1e-5—salltitles -b 60.0”. Other ncRNAs were filtered based on hits to Rfam CMs using Infernal (INFERence of RNA Alignment) tools described above. Finally, clean RNAseq reads were mapped to transcripts, and only transcripts with transcripts per million (TPM) values between 3 and 2000 were retained.

Prediction of tRNAs and rRNA was conducted using tRNAscan-SE 2.0.9 [65] with standard parameters:—thread 40 -qQ—detail -o results.txt -m stats.txt -j tRNA.gff3 -b tRNA.bed -a tRNA.fasta -f structures.txt -l log.txt -c /path/to/tRNAscan-se-2.0/bin/tRNAscan-SE.conf -s isospec_results.txt assembled_genome.fasta and Barrnap 0.9 (https://github.com/tseemann/barrnap) with options “—kingdom euk—threads 40”, respectively.

### Genome-wide identification of genes coding for Toll-like receptor (TLR)

All protein-coding genes predicted in this study were used for searching the presence and location of conserved protein domains using the InterProScan5 method [60] and 12 databases (CDD, Coils, Gene3D, MobiDBLite, PANTHER, Pfam, PIRSF, PRINTS, SMART, SUPERFAMILY, SignalP_EUK, and TMHMM) with default settings (e.g., CDD: e-value < 0.01). To minimize errors, Toll-like receptors (TLRs) were checked manually and confirmed for the presence of leucine-rich repeat (LRR) domain and toll/interleukin-1 receptor (TIR) domain that could contribute to a complete TLR structure using Apollo Annotation Editor [66]. Phobius was used to detect the regions of signal peptide (SP) and transmembrane (TM) regions [67]. Generally, a protein sequence that consists of one or more LRR motifs in the ectodomain (ECD) and TIR domains, joined by a single transmembrane helix, is considered a TLR. A complete TLR gene should contain an ATG start codon (methionine, M) and a stop codon, otherwise it is a partial TLR. According to the number of the C-terminal end of LRRs (LRRCT), TLRs can be classified into two categories: protostome-type (P-type, also known as mccTLR), and vertebrate-type (V-type, also known as sccTLR). P-type TLRs have a single cysteine cluster at LRRCT, while V-type TLRs have multiple cysteine clusters at LRRCT and sometimes even at the N-terminal end (LRRNT). P-type TLRs only exist in invertebrates; however, all vertebrate TLRs and some invertebrate TLRs belong to the V-type [41,45].

All the identified TLRs in this study were compared to TLR homologs reported in *B*. *glabrata* [27,34] using BLASTp and an E-value cutoff ≤ 1e-10. Proteins with ≥ 80% amino acid identity, ≥ 40% alignment coverage of the shortest protein, and a BLAST score ≥ 100 were considered as homolog. Amino acid (aa) sequences of the TIR domains extracted from TLRs and full-length TLRs identified from the iM line snail genome were aligned using MAFFT and converted into phylip 4 file format using BioEdit software (https://bioedit.software.informer.com/). A maximum likelihood (ML) tree was constructed using IQ-TREE [68] with standard model selection and 1,000 bootstrap replicates. The best-fit substitution model was determined using ModelFinder based on the minimum Bayesian information criterion value. The phylogenetic tree was displayed using the online tool iTOL (Interactive Tree of Life, https://itol.embl.de/). Gene Structure View (advanced) in TBtools software platform [69] was applied to visualize and edit the phylogenetic tree, protein domain architecture, and distribution of TLRs on the 18 chromosomes.

### Genome-wide identification of genes coding for fibrinogen domain containing proteins (FReDs)

Searching for conserved fibrinogen (FBG) domains was conducted similarly to that of TLRs described above. All the FReD identified in the studies were subjected to compare with FReD sequences reported in *B*. *glabrata* [55]. The criterion for FReD homologous protein search is the same as that of TLRs as described above. A complete *FReD* gene should contain an ATG start codon and stop codon, otherwise considered a partial gene. *Biomphalaria*-specific IgSF domains were predicted by a *B*. *glabrata*-specific HMM model with a minimum length of 40 aa and an E value of 0.00165 [55]. SP, N-terminal immunoglobulin superfamily (IgSF) domain(s), and C-terminal FBG domain that contribute to a complete FREP structure were determined in the same manner as that described in identification of conserved domains in TLRs above.

To examine the phylogenetic relationship among IgSF domains, FBG domain, and FReDs in the iM line snail genome, aa sequences of the IgSFs, FBGs, and full-length FReDs were used. Alignment of the sequences, construction of phylogenetic trees, and visualization of generated data were done in the same manner as described in TLRs above.

## Results

### Genome sequencing and assembly

**Fig 1:**
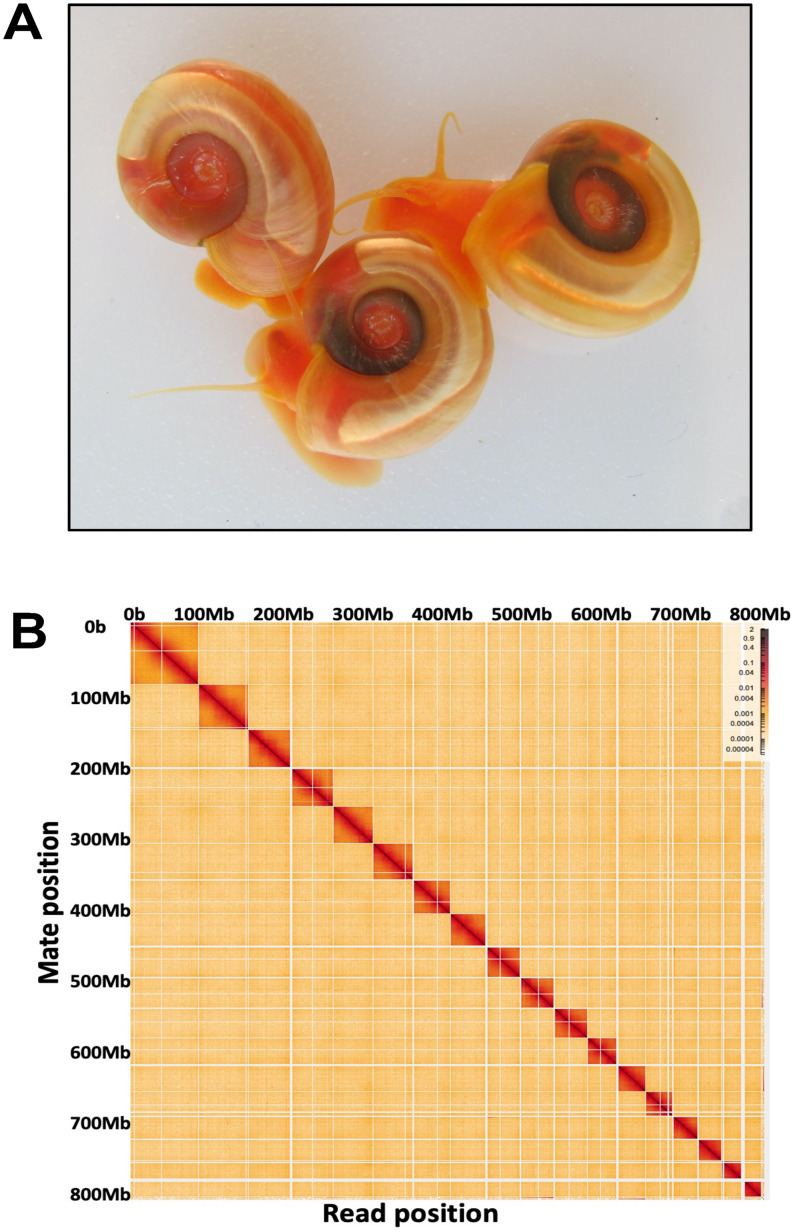
**A**) The image shows the iM line of *B*. *glabrata* snails used for genome sequencing. The photo was taken by S-MZ. **B**) The interaction heatmap shows 18 genome sequence contact matrices, representing 18 haploid chromosomes of *B*. *glabrata* (2n = 36). Colored dots indicate log formatted binned contact numbers (read pairs in Omni-C data). The dark-colored blocks marking the longest scaffolds correspond to the 18 chromosomes. Assembly of scaffolds at chromosome level was obtained from 120x PacBio continuous long reads (CLR), 150 x Illumina paired-end reads, and 74 x Omni-C proximity ligation reads.

The current chromosome-level assembled genome was constructed using the data produced from Illumina, PacBio, and Omni-C sequencing, generating 123,239, 100,217, and 60,837 Mb (million base) nucleotide sequences, respectively (Table 1). All these sequences generated from the homozygous iM line of *B*. *glabrata* ([34]; Fig 1A) resulting in 337x genome coverage. The initial assembly of the combined data generated from Illumina and PacBio yielded 255 scaffolds. Upon incorporating Omni-C reads, the existing scaffolds were further scaffolded, resulting in 18 sequence contact matrices representing 18 chromosomes (2n = 36) (scaffold N50 = 19.40 Mb) (Fig 1B). We designated these 18 matrices as 18 chromosomes based on their physical lengths and sizes (total number of nucleotides), ranging from the largest to the smallest (i.e., chromosome 1 to 18). Approximately 96.5% of genome sequences were anchored to these 18 chromosomes. The remaining 3.5% of sequences were not assigned to the chromosomes because they did not meet the criterion of proximity ligation of Omni-C data based on HiRise pipeline analysis [49]. The estimated size of the haploid genome of *B*. *glabrata* was estimated to be 842,576,133 bp (base pair). Completeness analysis showed that BUSCO based on Metazoa datasets (N = 954) revealed 96% of core genes, including complete single copy and duplicated ones.

**Table 1:** Overall statistics of the chromosome-level assembled genome.

Category	Metric	Value
Sequencing	Illumina (Mb)	123,239
	PacBio (Mb)	100,217
	Omin-C (Mb)	60,837
Assembly	Assembled length (bp)	842,576,133
	Number of scaffolds	18
	Mean scaffold length (bp)	46,809,785
	Longest scaffold length (bp)	90,496,816
	Shortest scaffold length (bp)	24,539,238
	Scaffold N50	19,395,504
	Scaffold L50	18
	GC content (%)	36
	BUSCO (%)	96
	Total length (> = 10 Kb)	842,576,133
	Number of contigs (> = 10 Kb)	18

Note: BUSCO analysis was performed based on OrthoDB v10 Metazoa datasets (N = 954, https://busco.ezlab.org/list_of_lineages.html).

**Fig 2:**
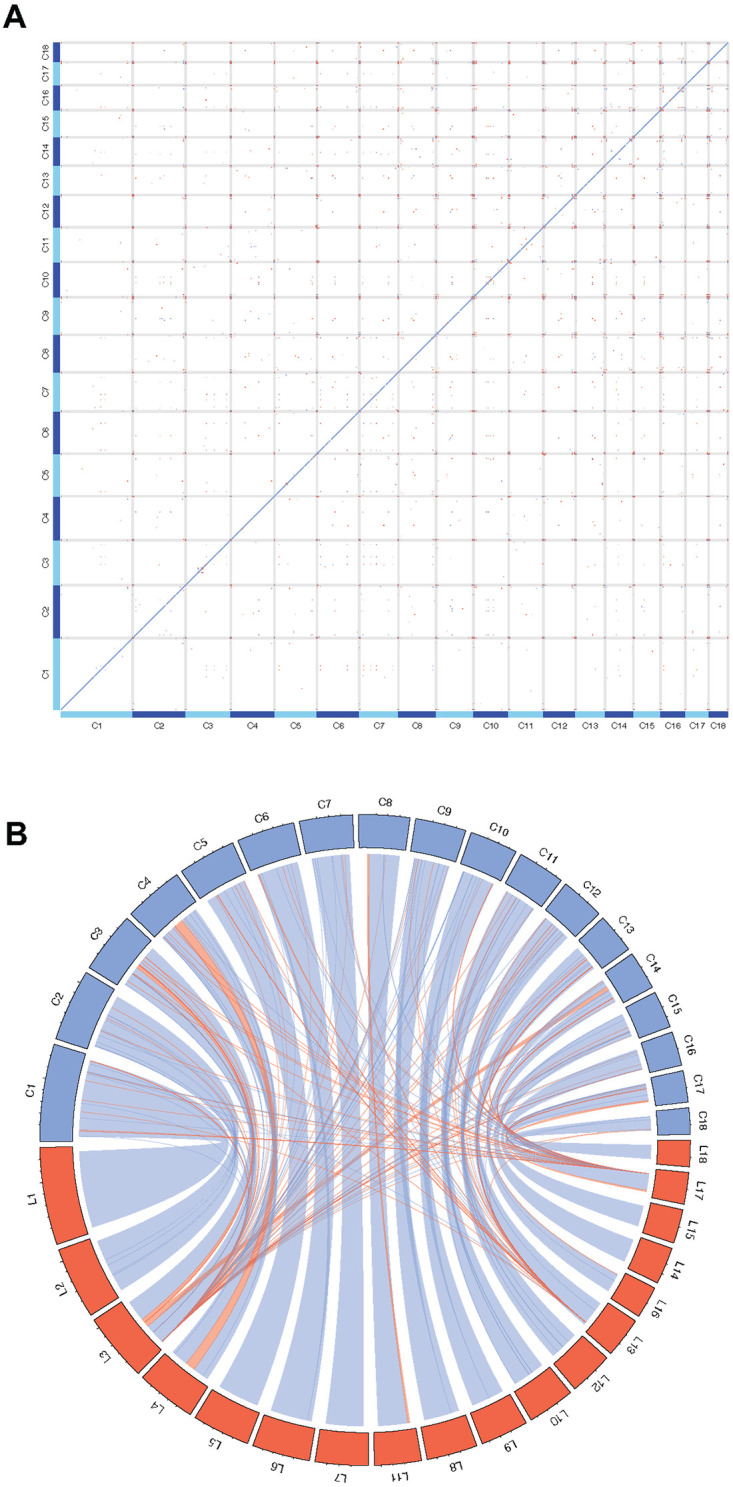
Synteny plot analyses show the comparison among the 18 chromosomes (**A**) and between the 18 chromosome-level assemblies and the 18 LGs (**B**). Blue and pink rectangles show matches in the same and reverse directions, respectively. The chromosome- and scaffold-level assemblies for the iM line of *B*. *glabrata* were compared at the nucleotide level using Minimap2.

Inter-chromosomal rearrangements or translocations in the *B*. *glabrata* genome were not detected (Fig 2A). Comparing the 18 chromosome assemblies to the 18 linkage groups (LGs) we previously reported [34] showed that all chromosomes can match a corresponding LG. The number assigned for a given chromosome may be different from that for LG because different methods were used for estimating the lengths. The length of LGs was calculated based on genetic distance whereas the size of chromosome (assembly) was based on the physical length of the DNA sequence (Fig 2B).

### Annotation of protein-coding genes, repetitive elements, and non-coding RNAs

**Fig 3:**
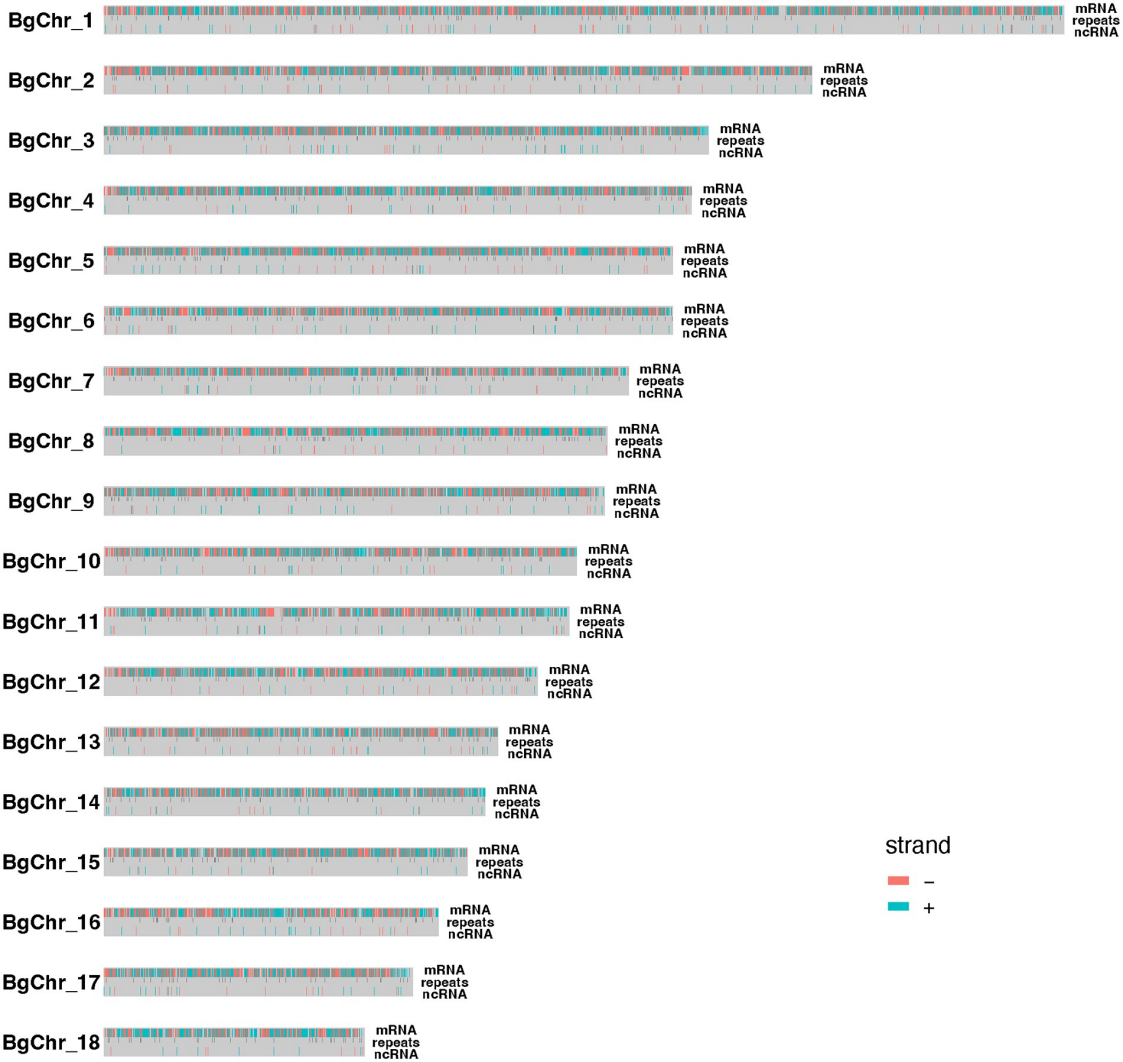
Density and distribution of protein-coding genes, repetitive elements, and non-coding RNAs on the 18 chromosomes.

A total of 34,559 protein-coding genes were predicted in the genome. Functional annotation was performed on the predicted gene models using the four major public databases, UniProt, NCBI non-redundant protein database (NR), NCBI non-redundant nucleotide database (nr), and InterProScan conservative domain database. Approximately 83% (28,702/34,559) of predicted gene models possess functional domains or have functional signatures. The four databases supported 14,107 protein-coding genes confirmed by the functional annotation (S1 Fig). At the chromosome level, the number of protein-coding genes in most chromosomes is generally correlated to the size of the chromosome. The highest and lowest number of genes were observed in chromosomes 1 (n = 3,633) and 18 (n = 968), respectively (Fig 3 and Table 2). Detailed information on gene models, including protein-coding genes and non-coding RNAs and functional annotations of gene models on the 18 chromosomes are provided in S1 Table.

**Table 2:** Chromosome-level genomic information.

BgChr	Length (Mb)	GC	Ns	Gene Models					
				Total	Coding	Non-coding			
					mRNA	miRNA	lncRNA	tRNA	rRNA
1	90.50	36.03%	100	3,913	3,633	9	247	24	0
2	66.75	36.17%	100	3,031	2,860	4	147	20	1
3	57.00	36.40%	400	2,753	2,591	3	144	15	125
4	55.38	36.23%	800	2,418	2,280	5	118	15	0
5	53.61	35.73%	200	2,562	2,384	8	162	8	1
6	53.60	36.23%	100	2,310	2,174	0	125	11	0
7	49.44	36.28%	0	2,129	2,020	6	97	6	0
8	47.42	36.06%	500	2,249	2,154	1	81	13	0
9	47.19	36.25%	200	1,975	1,852	3	103	17	1
10	44.57	36.43%	200	1,836	1,719	3	107	7	0
11	43.83	36.13%	300	1,676	1,556	3	102	15	0
12	40.86	36.27%	200	1,783	1,690	4	74	15	0
13	37.11	36.07%	400	1,585	1,474	9	98	4	0
14	35.91	35.92%	200	1,580	1,452	7	102	19	1
15	34.26	36.49%	100	1,439	1,354	0	77	8	0
16	31.52	36.15%	100	1,239	1,152	3	76	8	0
17	29.10	36.32%	300	1,333	1,246	3	75	9	2
18	24.54	36.41%	0	1,023	968	4	48	3	0
Total	842.59	36.20%	4,200	36,965	34,559	75	1,983	217	131

Note: Gene model includes mRNA and non-coding RNA; Chr: chromosome; Ns: numbers of unknown/undetermined nucleotide.

Repetitive sequences constitute a significant portion of the genome, contributing to 42.52%, with the majority being interspersed repeats (39.48%). The breakdown of repetitive elements reveals that 21.45% are retroelements, encompassing LINEs (14.78%), RET/Bov-B (8.42%), LTRs (6.67%), L2/CR1/Rex (4.28%), and Gyps/DIRS1 (3.42%). DNA transposons (3.23%) primarily consist of hobo-activator (2.93%), Tc1-IS630 (0.06%), and PiggyBac (0.05%) (refer to Fig 3 and Table 3 for details). Detailed information on the distribution of repetitive elements across the 18 chromosomes is also available (S2 Table).

**Table 3:** Repetitive elements identified in the *B*. *glabrata* genome.

Class	Sub-class	Number of elements*	Total length (bp)	Percentage of the genome
Retroelements		606,338	180,767,556	21.45%
	SINEs:	-	-	0.00%
	Penelope	206	75,580	0.01%
	LINEs:	498,142	124,528,212	14.78%
	CRE/SLACS	-	-	0.00%
	L2/CR1/Rex	159,526	36,099,949	4.28%
	R1/LOA/Jockey	4,505	5,023,285	0.60%
	R2/R4/NeSL	6,820	2,737,926	0.32%
	RTE/Bov-B	294,635	70,944,043	8.42%
	L1/CIN4	1,187	594,851	0.07%
	LTR elements:	108,196	56,239,344	6.67%
	BEL/Pao	-	-	0.00%
	Ty1/Copia	316	349,756	0.04%
	Gypsy/DIRS1	32,355	28,785,607	3.42%
	Retroviral	-	-	0.00%
DNA transposons		133,439	27,210,784	3.23%
	hobo-Activator	120,466	24,722,025	2.93%
	Tc1-IS630-Pogo	2,303	471,946	0.06%
	En-Spm	-	-	0.00%
	MuDR-IS905	-	-	0.00%
	PiggyBac	1,087	434,394	0.05%
	Tourist/Harbinger	-	-	0.00%
	Other (Mirage, P-element, Transib)	258	102,129	0.01%
Rolling-circles		257	147,833	0.02%
Unclassified		705,252	124,683,817	14.80%
Total interspersed repeats			332,662,157	39.48%
Small RNA		-	-	0.00%
Satellites		-	-	0.00%
Simple repeats		332,064	21,807,215	2.59%
Low complexity		44,411	3,624,601	0.43%

Notes:

* most repeats fragmented by insertions or deletions have been counted as one element

RepeatMasker version 4.1.1., default mode, run with rmblastn version 2.10.0+. Total sequences: 18, total length: 842,576,133 bp (842,571,933 bp excl N/X-runs). GC level: 36.18%.

For the 4 major non-coding RNAs, the number of miRNA, lncRNA, tRNA, and rRNA at the whole genome level is 75, 1,983, 217, and 131, respectively. The non-coding RNAs are randomly distributed on the chromosomes except for chromosome 3, which has 125 rRNAs (a total of 131 rRNAs in the genome) (Fig 3 and Tables 2 and S1).

### Identification and genomic organization of *TLR* genes

A total of 70 *TLR*-like genes were identified in the iM line of *B*. *glabrata* genome. All TLRs share a conserved ectodomain (ECD) composed of leucine-rich repeat (LRR) motifs, transmembrane (TM) segment(s), and a single cytoplasmic Toll/IL-1 receptor domain (TIR) except for BgiM30996-RA that does not have TM (S3 Table). LRRs ranged from 1 to 26 with a median of 9 LRRs per TLR. Double TMs were noted in eight TLRs, seven located at the front of the proteins and one at the end of the proteins. Single LRRCT (sccTLR) was found in 90% (63/70) of TLRs, whereas double LRRCTs (mccTLR) were observed only in one TLR (BgiM00446-RA). 74% (52/70) of TLRs are associated with LRRNT (S3 Table).

**Fig 4:**
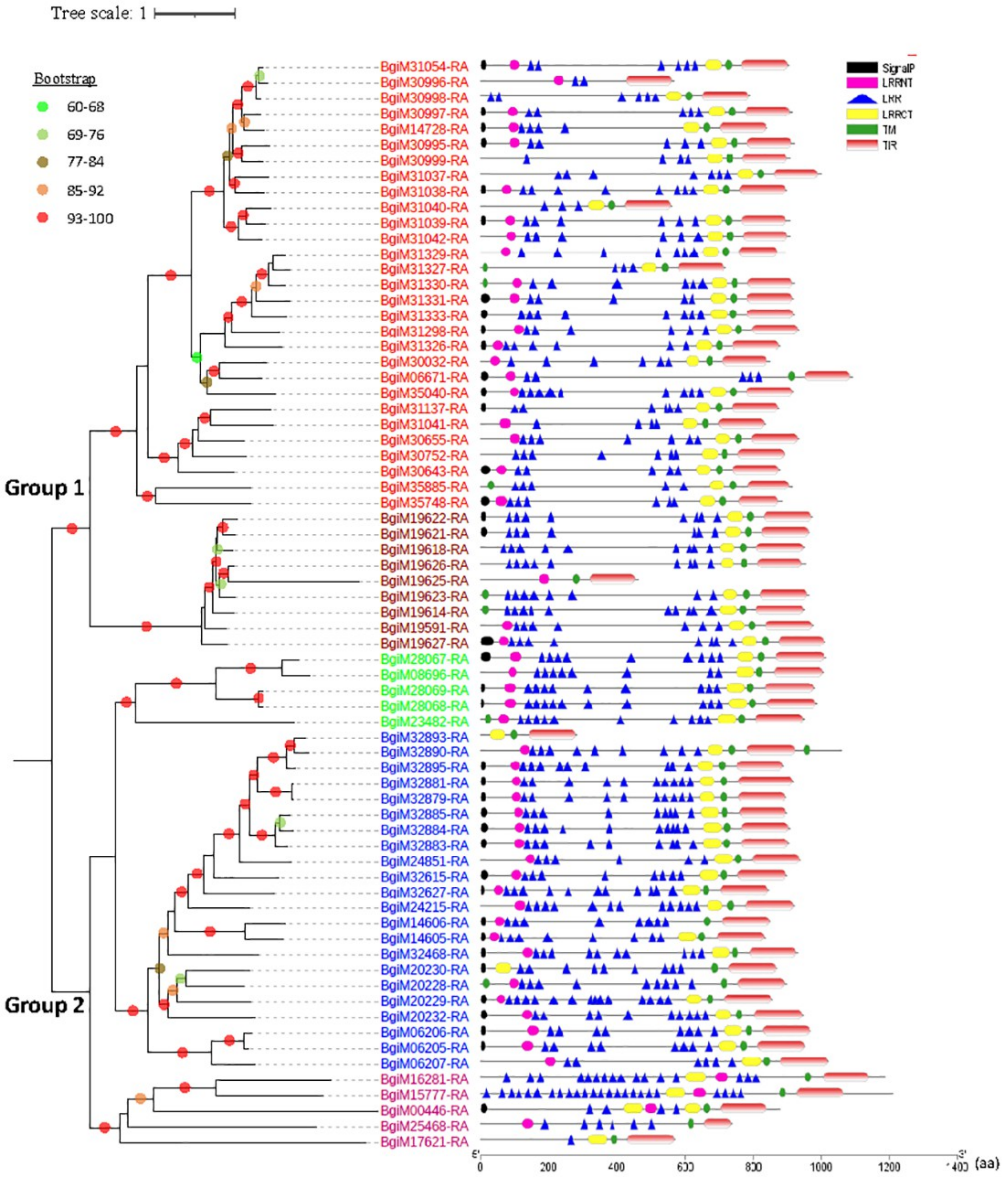
Phylogeny and domain structure of TLRs in the *B*. *glabrata* genome. Left panel: The ML tree was constructed with 1,000 bootstrap replicates using full-length protein sequences of 70 TLRs. ModelFinder selected WAG+F+R6 as the best-fit model for tree inference (Bayesian Information Criterion). Nodes with bootstrap support of 60 or higher are marked with different colors. Right panel: the structure of TLR gene products. TIR: Toll/interleukin-1 receptor; LRR: leucine-rich repeat; TM: transmembrane. LRRCT and LRRNT denote cysteine flanking regions of LRR at C- and N-terminus, respectively.

Phylogenetic analysis of the conserved TIR domains revealed seven distinct phylogenetic clusters derived from two major groups [27], with medium to high bootstrap support of >60% (S2 Fig). Group 1 contains all the previously identified TLRs in classes 1–3 except for BgTLR54 (BgiM24851-RA in Group 2) and a new cluster consisting of 9 TLRs. Group 2 has all the previously identified TLRs of classes 4–7 [27]. A similar pattern was observed in the phylogenetic tree using full-length TLRs (Fig 4).

**Fig 5:**
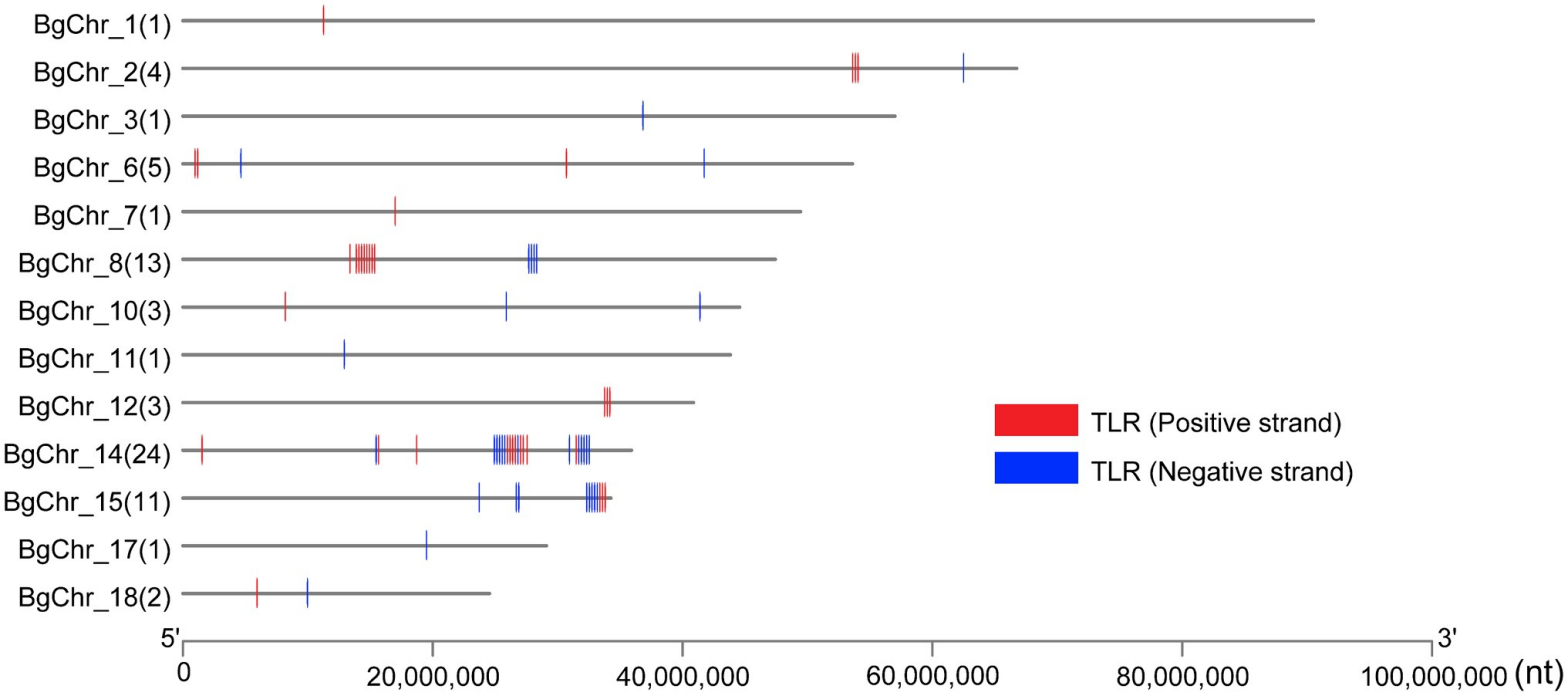
Distribution of *TLR* genes on *B*. *glabrata* chromosomes. The number in parenthesis is the number of *TLR* genes identified on the chromosome. Only chromosomes possessing *TLR* are shown.

The examination of genomic organization uncovered the dispersion of 70 TLR-like genes across 13 out of the 18 chromosomes (Fig 5). Chromosome 14 has the most significant number of *TLRs* (34.3%, 24/70), followed by chromosome 8 that contained 18.6% of the TLRs (13 out of 70), and chromosome 15 exhibited a notable presence with 15.7% (11 out of 70). In contrast, the remaining chromosomes each hosted 1 to 5 TLRs, collectively constituting 31.4% of the total TLRs.

### Identification and genomic organization of *FReD* genes

A total of 80 *FReD* sequences were identified in the iM line genome. Out of these, 40 FReDs share characteristics of FREPs (IgSF(s) + FBG), including 31 complete gene sequences and 9 partial genes. A total of 68 IgSF domains were revealed from the 40 FREPs. Eleven tropomyosin domains and 9 EGF (epidermal growth factor)-like domains were uncovered in the FReDs. The tropomyosin domain was exclusive to FREPs at the position between IgSF and FBG, previously called the Interceding Region (ICR). Among 9 EGF domains, only one was noted in FREP (BgiM29262-RA), and all others were associated with non-FREP FReDs (S3 Table).

**Fig 6:**
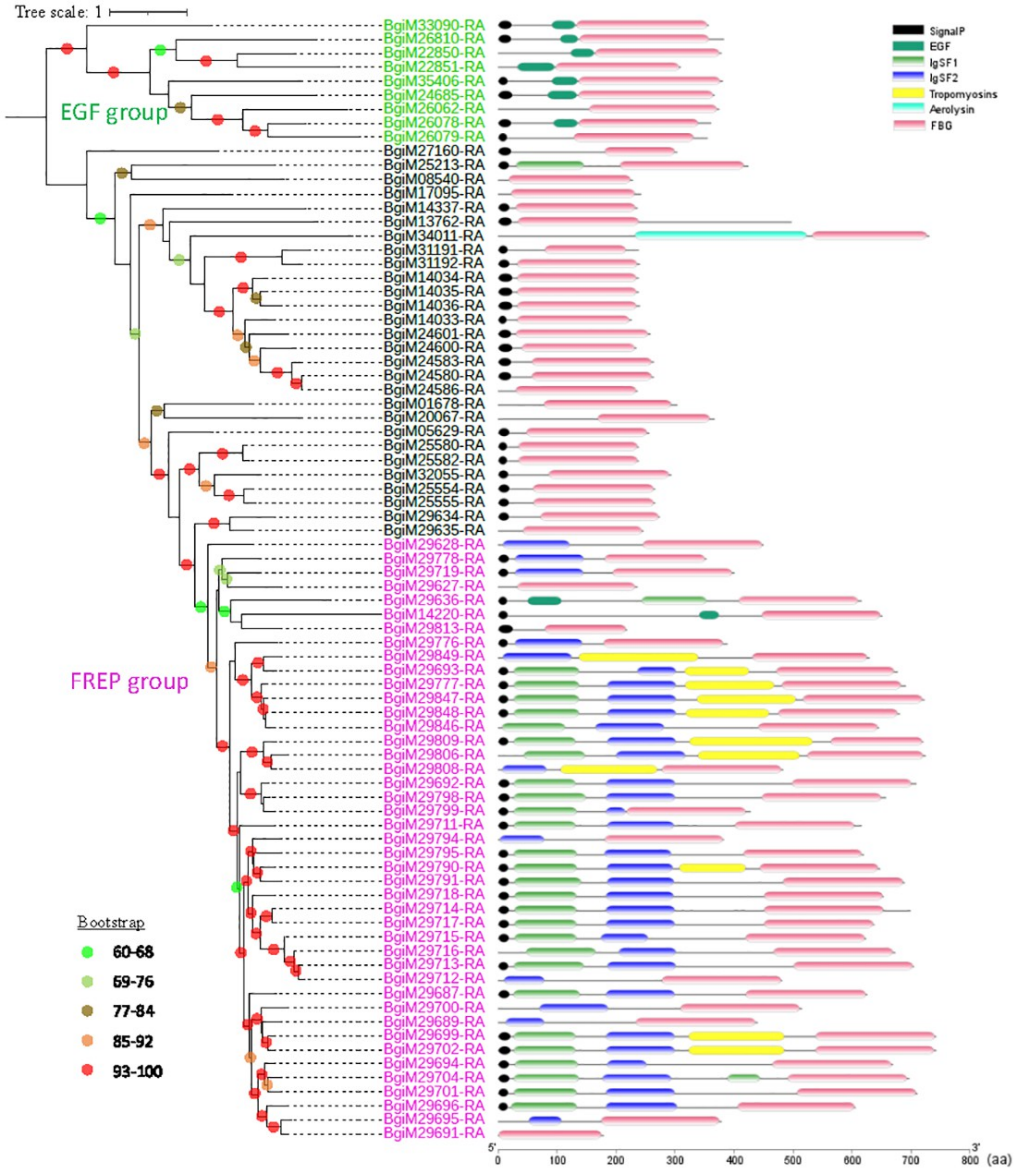
Phylogeny and domain structure of FReDs in the *B*. *glabrata* genome. Left panel: A ML tree was constructed using full length aa sequences of 80 FReDs. ModelFinder selected WAG+F+R7 as the best-fit model for tree inference (Bayesian Information Criterion). A test of 1,000 bootstrap replicates was performed and nodes with bootstrap support of 60 or higher are marked with different colorss. Right panel: structure of FReD gene products. EGF: epidermal growth factor; IgSF: immunoglobulin superfamily; FBG: fibrinogen domain.

Phylogenetic analyses revealed that almost all FREPs were grouped together (Figs 6 and S3). Specifically, within FREPs, the two types of IgSFs (IgSF1 and 2) were observed to form two distinct cluster groups (S3 Fig). FREPs, ficolin-like FReDs (FBG only), and EGF-containing FreDs, collectively referred to as FReM [70], were found to be distinctly separated (Fig 6). This separation suggests that *FREPs*, *FReM*, and *ficolin-like FReD* may have undergone different evolutionary histories.

**Fig 7:**
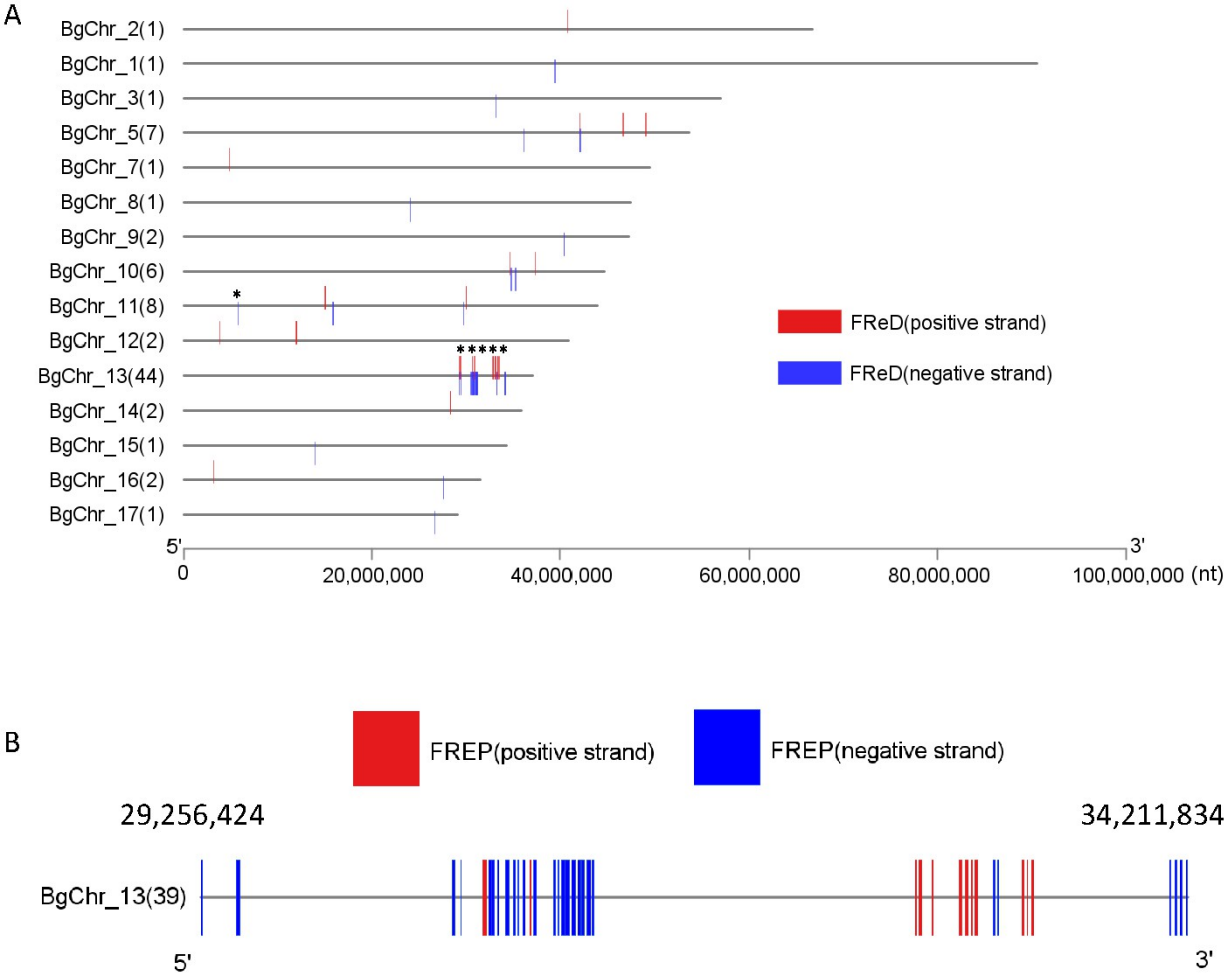
**A**) Distributions of *FReDs* on 15 chromosomes of *B*. *glabrata* and **B**) Distributions of 39 *FREPs* on chromosome 13 (location: 29256424–34211834). The number in parenthesis is the number of genes identified on the chromosome. * indicates the location of *FREPs*.

**Fig 8:**
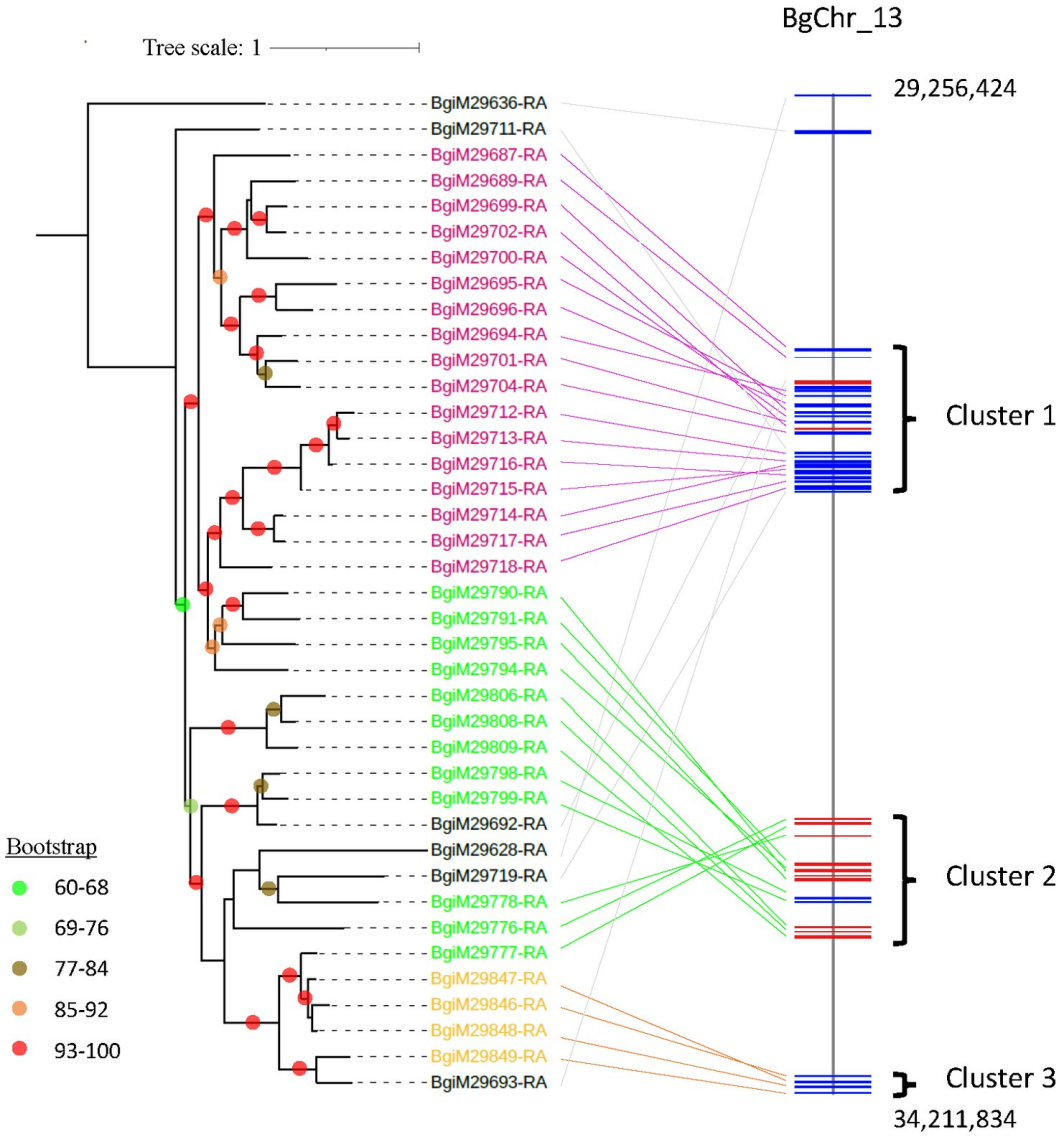
Phylogenetic relationship of 39 *FREPs* and their locations on chromosome 13. The ML tree was constructed with 1,000 bootstrap replicates. ModelFinder selected JTT+F+R4 as the best-fit model for tree inference (Bayesian Information Criterion). A test of 1,000 bootstrap replicates was performed and nodes with bootstrap support of 60 or higher are indicated by different colors *FREP* genes are grouped into 3 large clusters, in which their sequences are indicated by purple, green, and orange lines, respectively.

The genomic analysis highlights the distribution of *FReDs* across 15 chromosomes, with notable variations in abundance. Chromosome 13 stands out as the predominant locus, harboring the highest number of *FReDs* at 55% (44 out of 80). Subsequently, chromosome 11, chromosome 5, and chromosome 10 follow, with proportions of 10% (8/80), 8.8% (7/80), and 7.5% (6/80), respectively. The remaining chromosomes collectively account for 1–2 *FReDs* each, constituting 18.8% of the total FReDs (Fig 7A). Remarkably, of the 40 *FREPs*, 39 *FREPs* are clustered within a ~5 Mb region on the chromosome 13 (BgChr13: 29256424–34211834) except for one *FREP* (BgiM25213-RA) located on chromosome 11 (Fig 7B). Further analysis revealed the 39 *FREP* genes are grouped into three clades, which are well correlated to their chromosomal locations (Fig 8).

## Discussion

The advancement of our understanding in biomedicine is increasingly dependent on comprehensive genomic resources. Achieving complete chromosome-length sequences has been a longstanding objective in genome assembly. Through the utilization of multiple sequencing technologies and a homozygous genetic resource, we have successfully obtained chromosome-length genome sequences for the iM line snails. Given that the iM line is derived from *B*. *glabrata*, this carefully assembled and annotated genome now serves as a high-quality reference, effectively representing the genome of the species *B*. *glabrata*. This achievement contributes significantly to enhancing the precision and reliability of genomic data, thereby bolstering research and applications in biomedicine.

The chromosome-level physical assembly in large was validated by our recently constructed genetic linkage map although some numbers assigned to the linkage groups (LGs) may differ from the numbers of chromosomes based on our current assembly (e.g., chromosome 8 = LG11) (see Fig 2B) [34]. The assembly was constructed from a homozygous iM line, represents a haploid version of the *B*. *glabrata* genome. This approach overcomes the challenges associated with generating complex haplotype sequences using haplotype-resolved genome sequencing technology [71], thereby enhancing the reliability of the genome sequence. In the past, the majority of nuclear genome sequences published were assembled from multiple heterozygous individuals, each having two different sets of haplotype chromosomes. The resulting consensus sequence, representing a species’ genome, is a mosaic that does not naturally exist since it combines sequences from numerous haplotypes, each with distinct nucleotide sequences. In the present study, the nucleotide sequences of homologous chromosomes within an individual or from different individuals are identical because the source DNA was collected from three iM line snails of *B*. *glabrata*. The iM line was selected from a single M line via 81-generation of self-fertilization and confirmed to be a homozygous line using Kmer analysis (see [34]). So, all individual snails from the homozygous iM line are expected to be genetically identical. Consequently, the whole genome sequence presented here is not only assembled at the chromosome level but also resembles a haplotype-like genome of *B*. *glabrata*.

In previous decades, molecular studies of vector snail biology have focused on protein-coding genes. However, the biological functions of non-coding RNAs and repetitive elements in *B*. *glabrata* remain largely unexplored, despite extensive studies in other organisms suggesting their crucial roles in various molecular processes. The current study not only updated the annotation of protein-coding genes and repetitive elements at the genome level and added such information to individual chromosomes but also provided the first dataset of non-coding RNAs in *B*. *glabrata* genome, which was not reported in previous versions of the genome [27,34]. This comprehensive update broadens our understanding of the genomic landscape, shedding light on previously overlooked elements that may contribute significantly to the molecular details of *B*. *glabrata* biology.

From now on, we can focus on individual chromosome sequences of interest rather than the whole genome. The chromosome-level genome sequence will make *B*. *glabrata* more valuable as a model snail species and offer an excellent basis for in-depth studies of snail biology, mechanisms of snail resistance to schistosomes, and snail-parasite interactions. It also largely provides a valuable resource for comparative genomics and evolutionary biology.

While the genome represents a significant achievement with its chromosome-level assembly, covering 96.5% of the *B*. *glabrata* genome sequence through 18 pseudo-chromosomes (with only 3.5% remaining unmatched), we acknowledge that further improvements are necessary. For the human genome, it took an additional 21 years to fully complete the genome sequence since the first draft genome was published in 2001 [72]. More data generated from PacBio HiFi, ultra-long DNA sequencing, or other emerging long-read sequencing technologies [73] are needed to further improve the assembly. Particularly for genomes with high repetitive content, like the *B*. *glabrata* genome where repetitive sequences constitute 42.52%, high-quality long reads are invaluable. To refine the annotation, we recommend employing advanced computational analyses, including tools such as Apollo [66], and manual annotation. This approach has proven effective in enhancing the genome of other organisms, as demonstrated in the case of the parasite *Haemonchus contortus* [74]. We strongly encourage scientists within the research community to contribute to the ongoing improvement of this genome by participating in the annotation process. Collaborative efforts will undoubtedly enhance the utility of this genomic resource for future research endeavors.

One of the critical motivations for studying the model snail *B*. *glabrata* is to understand the fundamental mechanisms of the snail’s defense against parasites, particularly schistosomes, to develop snail-targeted biocontrol programs (i.e., blocking schistosomiasis transmission at the intramolluscan stage). We took advantage of the new genomic resource we report here to investigate the genomic structure of *TLR* and *FReD* gene families. These two families have been unequivocally established to play a crucial role in the immunity of invertebrates, including the gastropod *Biomphalaria* snails [40–46].

TLRs serve as pattern recognition molecules within Toll signaling pathways, which have been intensively investigated over the past two decades [40,45,75,76]. In *B*. *glabrata*, it has been demonstrated that RNAi-mediated knockdown of a mccTLR gene (Accession number AGB93809, corresponding to BgiM15777-RA in this study) in schistosome resistant BS90 of *B*. *glabrata* has resulted in a significant alteration of the resistant phenotype. Approximately 43% of BS90 snails, following RNAi-knockdown of the targeted TLR gene, exhibited the shedding of cercariae. This observation underscores the vital role of TLR-mediated pathways in the defense mechanisms of gastropod snails [40–46].

Using the new genomic resource, we identified 70 *TLR*-like sequences (62 complete *TLR* genes) and provided detailed information regarding their genomic locations, orientation, and phylogenetic relations. A previous study revealed 56 *TLR*-like sequences (27 complete *TLR* genes) in the BBO2 strain of *B*. *glabrata* genome [27]. Nevertheless, a large number of *TLRs* uncovered in the *B*. *glabrata* genome further supports the notion of an expansion of *TLRs* in the phylum Mollusca although the reason for such a massive expansion is still unknown [45].

We mapped all 70 *TLR* genes to the *B*. *glabrata* chromosomes and elaborated the phylogenetic relations among all the members. The phylogenetic tree revealed the presence of a new clade of TLRs, which comprises 9 members. Furthermore, we found 8 TLRs possessing double transmembrane (TM) segments, here designated as two-TM-TLR. The presence of two TMs in a TLR is not common, but was observed in mammals [77], Asian seabass [78], and the Manila clam *Ruditapes philippinarum hepatopancreas* [79]. A functional study has shown that infection of the Gram-negative bacterium *Vibrio anguillarum* can increase expression of a *two-TM -TLR* gene in the Manila clam [79]. The biological implications of the two-TM-TLRs in *B*. *glabrata* are currently unknown.

For FReDs, many members such as ficolins (FBG domain only) and FREPs (IgSF(s) + FBG) have been confirmed to play a crucial role in defense in both vertebrates and invertebrates [42,44,46]. A previous study based on the BBO2 genome revealed 73 *FReD* genes. Of these 73 *FReD*, 39 are *FREPs*, which include 26 complete and 13 partial sequences [55]. Current chromosome-level assembly enabled us to identify 80 *FReD* genes, half of which are *FREPs*, further improving our understanding of FReD diversity in *B*. *glabrata*.

In addition to adding new FReD members, we discovered several novel members consisting of domains or domain combinations that were not previously described. Among the 40 FREPs, 11 FREPs possess a tropomyosin domain at the position between IgSF and FBG. The absence of tropomyosin in previous descriptions of FREPs could be attributed to 1) the oversight in detecting FREPs containing tropomyosin in earlier studies, and 2) the utilization of advanced bioinformatics tools and multiple databases in our analysis. Tropomyosin is an important component of the muscular system working in conjunction with troponin to regulate muscle contraction [80]. An interesting question is whether integration of the tropomyosin domain in some FREPs may lead to a function different from those FREPs lacking tropomyosin. Furthermore, we found new domain combinations, which include EGF + IgSF + FBG (BgiM29636-RA) and triple IgSF + FBG (BgiM29704-RA).

Of the 80 *FReD* genes, 40 non-*FREP’s FReD* (without IgSF) are distributed across 15 chromosomes. The distribution pattern is very much like that of *TLR* gene families, dispersing on 13 chromosomes. Surprisingly, almost all *FREPs* (39 of 40) are clustered in a small genomic region (~5 Mb) of chromosome 13 (with a size of ~37 Mb). In the past years, we have made considerable efforts to understand FREP biology, discovered somatic diversification of the *FREP3* gene via hypermutation and gene conversion, and revealed their anti-schistosome function [47,48,70,81–86]. The diversification of immune genes has been documented in various invertebrates in recent years. However, the underlying mechanisms, including those for *FREPs*, remain largely unknown [87–93]. We hypothesize that the clustering of a substantial number of closely related *FREP* genes in a small genomic region may promote the exchange of genetic material (DNA) between *FREP* genes, thus increasing their diversity. Further investigations are needed to focus on this 5 Mb region, which may help decipher the diversification mechanism of *FREPs* in *B*. *glabrata*.

We acknowledge the existence of a chromosome-level assembled and annotated genome of *B*. *glabrata* (strain unknown) deposited in the NCBI database (referred to as NCBI data, specifically xgBioGlab47.1) produced by the Wellcome Sanger Institute Tree of Life Programme (www.sanger.ac.uk/programme/tree-of-life/). In our comparative analysis with this dataset, the overall quality of our genome, derived from the unique iM line, is largely independently confirmed by the NCBI data. It’s important to note that the two datasets were generated using different sequencing methods and snail strains. The total nucleotide length of our assembly is 842,576,133, while the NCBI assembly is 845,861,586. These values significantly differ from the initial draft of the *B*. *glabrata* genome (916 Mb) [27]. Several key features exhibit high similarity between our dataset and the NCBI data, including N50 (49,437,159 vs. 48,536,009), mean length (46,809,785 vs. 46,992,310), and longest (90,496,816 vs. 90,404,365) and shortest contigs (24,539,238 vs. 24,813,541), suggesting the two assemblies are of similar quality. Compared to the NCBI data, our genome has fewer gaps (42 vs. 157) and a lower count of unknown nucleotides (4,200 vs. 31,400). Importantly, our haplotype-like genome, constructed with higher genome coverage (337x vs. 29x), was generated from a homozygous iM line—a model that has recently found applications in genetic linkage mapping, quantitative trait locus (QTL) analysis, genomics, and immunological studies (see [34]). The M line from which iM was developed is the best studied snail model that has been used for schistosomiasis research since 1950’s [17]. The fundamental molecular and genomic knowledge gained from these two lines can be seamlessly transferred for further research. Therefore, the current haplotype-like, chromosome-level assembled, and annotated genome of *B*. *glabrata* serves as an outstanding genomic resource for advancing studies on schistosomiasis, one of the world’s most significant neglected tropical diseases.

## Conclusion

Our study achieved two significant steps. Firstly, we successfully generated a chromosome-level assembled and annotated genome of the gastropod snail *B*. *glabrata*, representing the first chromosome-level genome of all schistosomiasis vector snails. Secondly, we provided a comprehensive genomic view of two crucial immune-related gene families (*TLRs* and *FReDs*), identified their new members, and uncovered a cluster of *FREP* genes. The genomic resource and findings presented in the paper will help better understand the biology of the vector snails, particularly the model snail *B*. *glabrata*.

## Supporting information

S1 Table Genomic location of all gene models predicted. (XLSX)

S2 Table Information of repetitive elements at the chromosome level. (XLSX)

S3 Table Genomic information of TLRs (a) and FReDs (b). (XLSX)

S1 Fig Functional annotation for predicted gene models in *B*. *glabrata*. Four major bioinformatics databases including Uniports database, NCBI-non-redundant nucleotide (NT) database, NCBI non-redundant protein (NR) database, and InterProScan integrated conserved database were applied. Percentages were calculated based on a total of 34,559 gene models.
(TIFF)

S2 Fig Phylogenetic relationship of TIR domains predicted from TLR genes in *B*. *glabrata*. A ML tree was constructed using amino acid (aa) sequences, with 1,000 bootstrap replicates. ModelFinder selected LG+G4 as the best-fit model for tree inference (Bayesian Information Criterion). Nodes with bootstrap support of 60 or higher are marked with different colors. Domains were labeled with the protein ID followed by the domain’s name. Asterisk (*) denotes a new cluster identified in current work.
(TIFF)

S3 Fig Phylogenetic relationship of FBGs (A) and IgSFs (B) derived from FReDs identified in *B*. *glabrata*. **A**) A ML tree was constructed using amino acid (aa) sequences, with 1,000 bootstrap replicates. ModelFinder selected WAG+F+R6 as the best-fit model for tree inference (Bayesian Information Criterion). Blue labels indicated those domains extracted from FREPs and red labels from non-FREPs. **B**) A ML tree was constructed using amino acid (aa) sequences, with 1,000 bootstrap replicates. ModelFinder selected JTTDCMut+G4 as the best-fit model for tree inference (Bayesian Information Criterion). Green and blue labels indicate IgSF1 and IgSF2, respectively. Nodes with bootstrap support of 60 or higher are marked with different colors. Domains were labeled with protein ID followed by the start position of the domain.
(TIFF)
