# Supplementary figures and images for: A haplotype-like, chromosome-level assembled and annotated genome of Biomphalaria glabrata, an important intermediate host of schistosomiasis and the best studied model of schistosomiasis vector snails
Source: PLoS Negl Trop Dis. 2024 Feb 29;18(2):e0011983. doi: 10.1371/journal.pntd.0011983 (PMC10903818; doi:10.1371/journal.pntd.0011983)

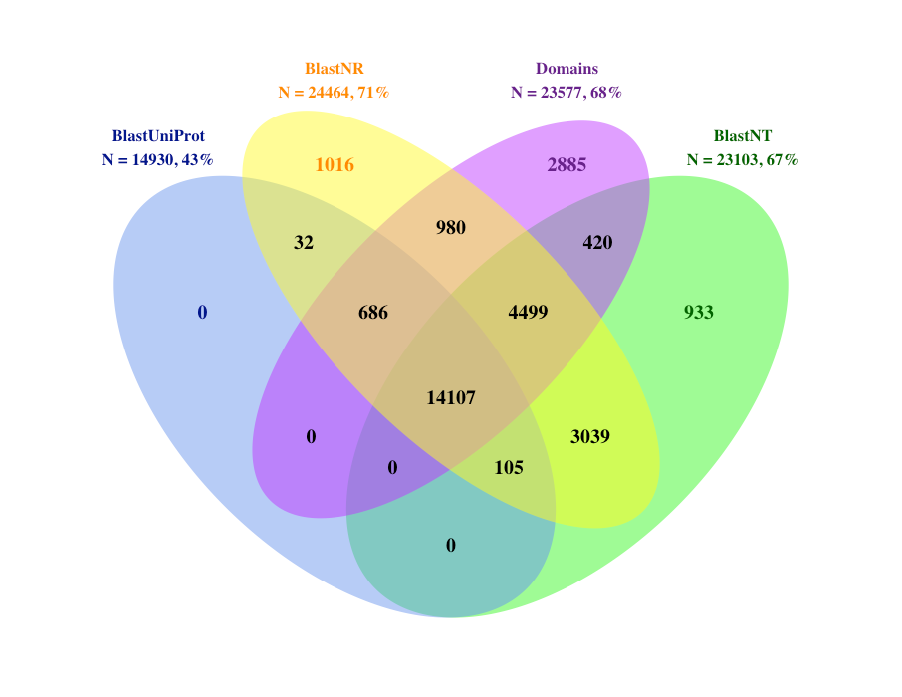

Supplement: S1 Fig — Four major bioinformatics databases including Uniports database, NCBI-non-redundant nucleotide (NT) database, NCBI non-redundant protein (NR) database, and InterProScan integrated conserved database were applied. Percentages were calculated based on a total of 34,559 gene models. (TIFF) [file pntd.0011983.s004.tiff]

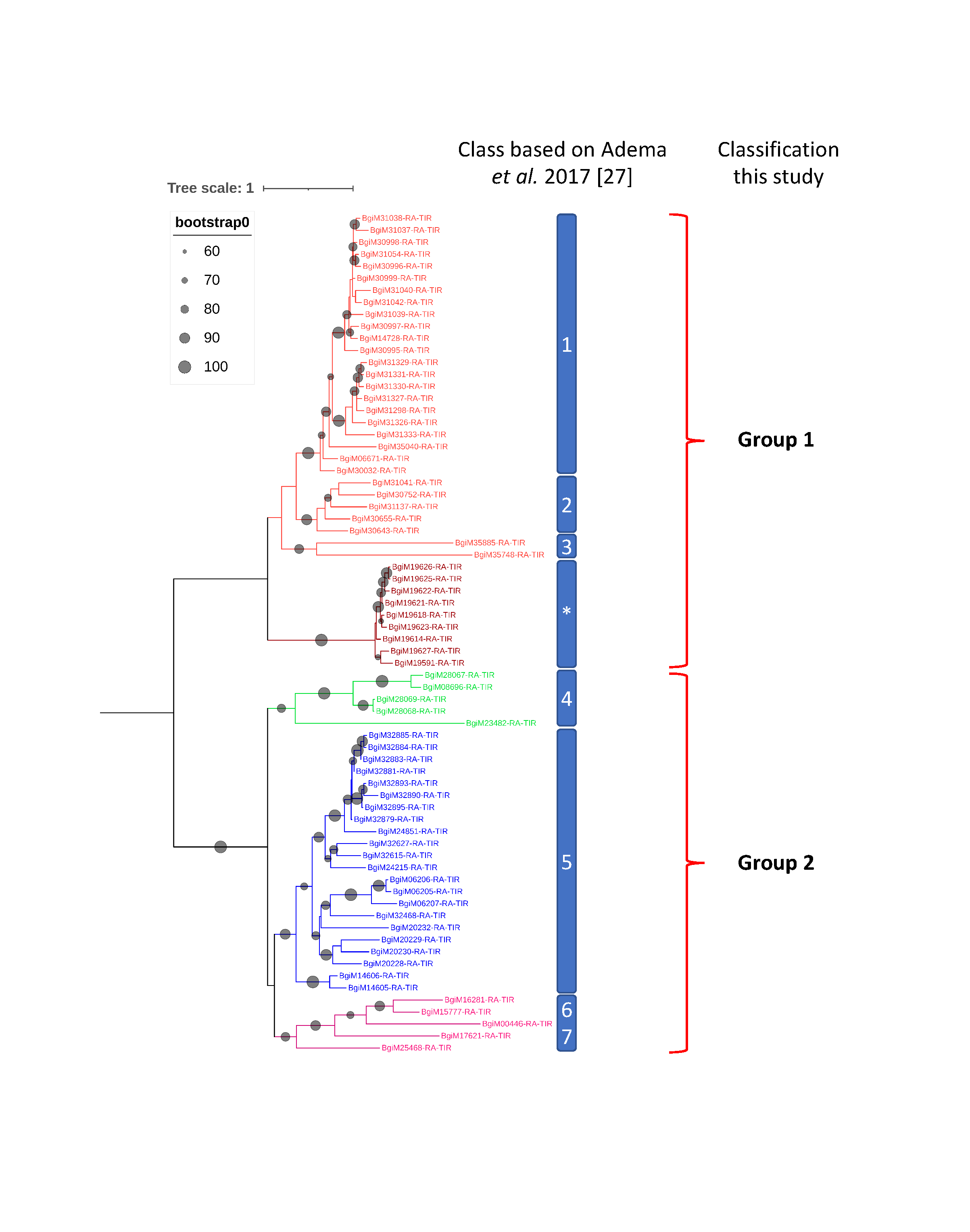

Supplement: S2 Fig — A ML tree was constructed using amino acid (aa) sequences, with 1,000 bootstrap replicates. ModelFinder selected LG+G4 as the best-fit model for tree inference (Bayesian Information Criterion). Nodes with bootstrap support of 60 or higher are marked with different colors. Domains were labeled with the protein ID followed by the domain’s name. Asterisk (*) denotes a new cluster identified in current work. (TIFF) [file pntd.0011983.s005.tiff]

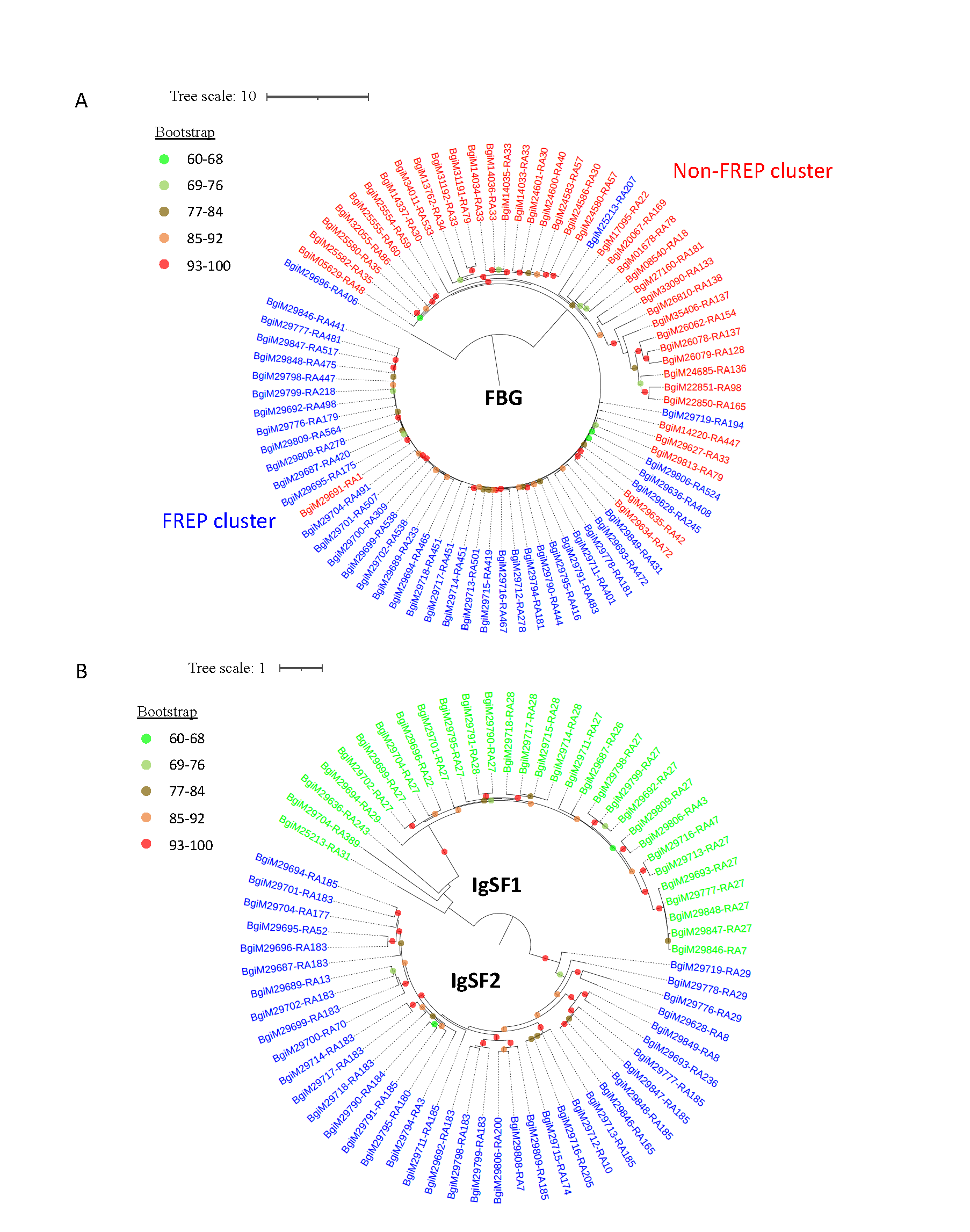

Supplement: S3 Fig — A) A ML tree was constructed using amino acid (aa) sequences, with 1,000 bootstrap replicates. ModelFinder selected WAG+F+R6 as the best-fit model for tree inference (Bayesian Information Criterion). Blue labels indicated those domains extracted from FREPs and red labels from non-FREPs. B) A ML tree was constructed using amino acid (aa) sequences, with 1,000 bootstrap replicates. ModelFinder selected JTTDCMut+G4 as the best-fit model for tree inference (Bayesian Information Criterion). Green and blue labels indicate IgSF1 and IgSF2, respectively. Nodes with bootstrap support of 60 or higher are marked with different colors. Domains were labeled with protein ID followed by the start position of the domain. (TIFF) [file pntd.0011983.s006.tiff]
